# Parent-mediated interventions versus usual care in children with autism: A systematic review with meta-analysis and Trial Sequential Analysis

**DOI:** 10.64898/2026.08.06.26357818

**Published:** 2026-08-10

**Authors:** Charlotte Engberg Conrad, Sonja Martha Teresa Ziegler, Niels Bilenberg, Jens Christiansen, Kirstine Agnete Davidsen, Birgitte Fagerlund, Emil Færk, Helle Jakobsen, Rikke Hermann Jakobsen, Pia Jeppesen, Caroline Kamp, Tina Røndrup Kilburn, Per Hove Thomsen, Manon Varenne, Martin Vestergaard, Janus Christian Jakobsen, Marlene Briciet Lauritsen

## Abstract

**Objectives:**

To evaluate the positive and adverse effects of parent-mediated interventions (PMIs) versus care as usual for children with autism.

**Setting:**

Systematic review and meta-analysis and Trial Sequential Analyses (TSA), following the Preferred Reporting Items for Systematic Reviews and Meta-Analyses guidelines.

**Methods:**

We searched for randomised clinical trials of PMIs for children with autism in the databases CENTRAL, EMBASE, LILACS, PsycINFO, MEDLINE, and SCI-EXPANDED (up to 13 August, 2025), complemented with manual searches. 12,359 articles were screened. Data were synthesised using meta-analyses and Trial Sequential Analyses (TSA), and risks of bias and certainty of the evidence were evaluated.

**Primary and secondary outcome measures:**

The primary outcome was autism characteristics. Secondary outcomes were adverse effects, child adaptive functioning, child language, child and parent quality of life, and parental stress. Ten exploratory outcomes were included.

**Results:**

32 trials (N=1,625) comparing PMIs to usual care, waiting list, or no intervention were included. All trials had a high risk of bias. The multiplicity-adjusted threshold for statistical significance was *p* = 0.013 due to the number of outcomes. Meta-analyses and TSAs showed it could be rejected that PMIs reduced autism characteristics (MD = −0.88; 95% confidence interval −2.92 to 1.15; *p* = 0.05, 4 trials, *N* =353, low certainty), child adaptive functioning (7 trials, *N* =408), child language (4 trials, *N* =308), or parental stress (7 trials, *N* =385). Due to insufficient data, the remaining secondary meta-analyses could not be conducted. Meta-analyses of exploratory outcomes showed beneficial effects concerning child behaviour problems and parent sensitivity/synchronicity.

**Conclusions:**

This meta-analysis found no benefits of PMIs on child autism characteristics, child adaptive functioning, child language, or parental stress. Benefits were found in reduction of child behaviour problems and improved parent sensitivity/synchronicity. The evidence remains uncertain, and more trials including outcomes of adverse effects and quality of life are needed.

**Trial registration:**

CRD42022385188 PROSPERO

## Full Text Availability

The license terms selected by the author(s) for this preprint version do not permit archiving in PMC. The full text is available from the preprint server.

